# Deliberate versus emergent organisational agility in paediatric health-care delivery: evidence from an Italian case study

**DOI:** 10.1108/JHOM-05-2025-0292

**Published:** 2026-05-29

**Authors:** Mario Casolino, Lavinia Benedetti, Francesco Venier, Martina Vardabasso, Grazia Garlatti Costa

**Affiliations:** Institute for Maternal and Child Health, IRCCS “Burlo Garofolo”, Trieste, Italy; Department of Economics, Business, Mathematics and Statistics “Bruno de Finetti” (DEAMS), MIB Trieste School of Management, Trieste, Italy

**Keywords:** Organisational agility, Lean healthcare, Dynamic capabilities, McKinsey 5S, Mixed methods, Paediatrics, Italy

## Abstract

**Purpose:**

This paper examines how organisational agility, defined as an institution's ability to thrive in an unpredictable and ever-changing business environment, presents itself within a pediatric day hospital, a little-explored public sector context, and how it influences the patient experience.

**Design/methodology/approach:**

We integrated (1) a real-time survey of 443 patient journeys, (2) a 109-respondent agility questionnaire for healthcare professionals and (3) 16 semi-structured interviews, following the McKinsey 5S framework and the dynamic-capabilities view. Descriptive statistics and ordinal-logistic models were triangulated with thematic coding.

**Findings:**

Agility scores were highest for structure (83%) and people (73%) but lowest for strategy (46%) and technology (40%). Flexible scheduling keeps median appointment deviations to 32minutes while sustaining high satisfaction (4.5/5). Staff tenure > 15 years predicts lower perceived agility (β = –0.27, *p* < 0.01). Transparent information and courteous interactions raised the odds of top-quartile patient experience more than eightfold (*p* < 0.001).

**Practical implications:**

A four-step roadmap (strategic alignment, user-centred IT, experiential learning loops, senior-staff engagement) can institutionalise “deliberate agility” in similar units.

**Originality/value:**

This is the first study to operationalise the 5S framework in an Italian tertiary paediatric context, offering a granular, data-driven link between agility and patient experience.

## Introduction

The healthcare sector increasingly faces the challenge of enhancing efficiency, reducing waste, and creating sustainable value for patients and providers (Modugno *et al*., 2024). In this context, agility and Lean management principles have emerged as strategic methodologies to streamline healthcare processes, reduce inefficiencies, and prioritise value-added activities.

In today’s increasingly complex and volatile healthcare environment, providers must be able to quickly adapt to technological advances, changing patient needs, regulatory evolutions, and sudden disruptions. Agility is not a one-time reaction but a continuous ability to anticipate and adapt to change in real time without compromising performance or service quality (Dove, 1999; Doz and Kosonen, 2010).

Therefore, organisational agility refers to an organisation’s characteristic to adapt quickly and effectively to internal and external changes while preserving its mission and core values (Teece, 2014). This adaptability is driven by decentralised structures, promoting rapid decision-making and innovation by enabling the distribution of decision-making rights across multiple levels and allowing teams to dynamically respond to emerging challenges without waiting for top-down approval (Teece, 2016).In healthcare settings, where timely decisions can directly influence patient outcomes (Gittell *et al*., 2020), organisational agility reflects the healthcare organisation's efficiency in prospering in unpredictable and continuously changing environments, while maintaining care quality and strategic alignment (Tolf *et al*., 2015).

Cross-functional teams comprising clinical staff, managerial figures, and IT specialists are central to this model. By collaborating across disciplines, they enhance communication, accelerate problem-solving, and facilitate workflow adaptation to emergent challenges (Highsmith, 2009; Aghina *et al*., 2017; Holbeche, 2022; Villa *et al*., 2009; Andreasson *et al*., 2016).

Organisations embracing agility promote continuous learning, data-driven feedback loops, and iterative refinement of practices in reply to real-time information, contributing to the quality, responsiveness, and sustainability of care (Denning, 2018; Sutherland *et al*., 2017). Constant improvement and agile management are essential for enhancing care quality and adaptability in healthcare systems (Berlin *et al*., 2017; Mandal, 2020; Tolf *et al*., 2015). Research reveals that agile institutions have a 70% chance of being in the top quartile of organisational health, which is considered the best indicator of long-term performance (Bazigos *et al*., 2015).

Despite its relevance, organisational agility remains underexplored in public-sector healthcare, particularly in high-complexity paediatric environments (Walter, 2021; Tolf *et al*., 2015; Ahmad and Wasim, 2023).

This study addresses that gap through an empirical case analysis of the Paediatric Day Hospital (DH) at the Institute for Maternal and Child Health–IRCCS Burlo Garofolo – Trieste, Italy.

The case is unique due to three key factors: the high organisational complexity of managing paediatric patients with rare and complex conditions, the necessity for coordination across multiple specialised departments, and the typical constraints of public healthcare systems, including limited human and financial resources (Plsek and Wilson, 2001; Stake, 1995).

This research explores how agility principles manifest in this context and how we can operationalise it according to McKinsey’s 5S Framework which encompasses strategy, structure, process, people, and technology (Aghina *et al*., 2017).

This case offers a key opportunity to understand how agile methodologies can be adapted and implemented in environments characterised by hierarchical structures and the dual need to standardise and customise services (Mintzberg, 1979; Eisenhardt, 1989; Flyvbjerg, 2006) despite the presence of well-documented challenges such as regulatory constraints, documentation burdens, resistance to change, and limited scalability in healthcare settings (Ahmad and Wasim, 2023).

To guide the inquiry, we pose three explicit Research Questions (RQs):

RQ1.
*Diagnosis:* What is the current level of agility across the 5S dimensions in a public paediatric DH?

RQ2.
*Mechanisms:* How do frontline micro-practices enable or hinder agility when strategy and technology are weak?

RQ3.
*Determinants:* Which organisational variables (e.g. professional role, tenure, age) significantly predict perceptions of agility?

## Theoretical background

Although agile methodology has been successfully applied in sectors such as finance, construction, and marketing, its adoption in healthcare remains limited and under-researched (Alotaibi and Almudhi, 2023).

Existing literature acknowledges its potential to improve delivery speed, service quality, and stakeholder satisfaction in clinical contexts (Improta *et al*., 2020); however, findings often lack empirical depth, mainly regarding patient-centred outcomes and satisfaction (Alotaibi and Almudhi, 2023).

Recent studies suggest that agile practices in healthcare can enhance collaboration, accelerate decision-making, and improve responsiveness in crisis scenarios (Nanji *et al*., 2018), yet integration with existing hierarchies and workflows remains challenging. Communication breakdowns, linked to over 60% of medical errors, have also highlighted the need for agile-enabled teamwork and stakeholder engagement (Ribeiro and Domingues, 2018). Moreover, the sector still lacks standardised metrics for assessing operational effectiveness and struggles with technology adoption, such as data interoperability, which agility can help overcome (Unterhofer *et al*., 2021). Despite early successes in healthcare IT and patient safety projects (Alotaibi and Almudhi, 2023), literature still underrepresents patient-centred outcomes, reinforcing the demand for empirical exploration of agility in complex paediatric settings.

Recent efforts to enhance hospital performance have focused on managing internal complexity through process improvement strategies, particularly Lean-influenced approaches, to improve care quality and patient safety (Kaplan *et al*., 2014). In healthcare, Lean eliminates non–value–adding activities to improve patient flow, information, or materials (Womack *et al*., 2005). This method has been successfully applied in hospital settings, positively influencing cost, quality, and satisfaction (Radnor *et al*., 2012); however, growing critiques suggest it narrowly targets technical improvements and internal efficiency, often overlooking external effectiveness and system-level responsiveness.

Although Lean and Agile methods are often viewed as distinct paradigms, several authors suggest they can be complementary, the first one providing structure and process discipline, and the second enhancing responsiveness and flexibility. Others argue that agile represents a new evolutionary stage beyond Lean, better suited to contexts of uncertainty and high variability. In practice, hybrid models increasingly combine the two approaches to balance efficiency with adaptability across varying scenarios (Li and Martins, 2024).

To address this gap, scholars have emphasised the need for more adaptive models, particularly agile approaches capable of handling both internal performance and external complexity (Radnor and Osborne, 2013). By enabling organisations to quickly adjust structures, processes, and strategies, agility is a complementary paradigm that may help hospitals go beyond Lean and build capabilities for environmental sensing, real-time adaptation, and cross-boundary coordination (Goldman *et al*., 1995; Tolf *et al*., 2015).

This study adopts the McKinsey 5S Framework to operationalise agility, which identifies five key dimensions that drive organisational agility: strategy, structure, process, people, and technology (Aghina *et al*., 2017). *Strategy* centres on shared purpose, referred to as the organisation's “North Star, and the flexible allocation of resources that fosters personal commitment and emotional engagement among staff members”.

The structure reflects cross-functional collaboration within flat hierarchies, where clearly defined and accountable roles are embedded in an open, trust-based environment that fosters shared responsibility and a high degree of autonomy.

The process emphasises rapid iteration, transparent practices, and a culture of continuous learning, enabling swift adaptation to change, real-time solution testing, and seamless feedback integration to support ongoing improvement and adaptive learning processes (Bergaoui and Ghannouchi, 2023).

The expectation is that people will embrace change, proactively contribute, and take ownership of team goals, fostering an adaptive leadership culture and a mindset essential to sustaining high-performance environments.

Technology serves as both an enabler and accelerator of organisational responsiveness. It acts as a catalyst to unlock value, accelerate innovation, and support agile decision-making by optimising processes and enabling rapid responses to evolving demands and stakeholder expectations.

This view also aligns with the Dynamic Capabilities Framework, which defines agility through three processes: sensing changes, seizing opportunities, and reconfiguring resources to meet evolving demands (Teece, 2014). In clinical contexts, these capabilities translate into modifying care protocols, reallocating teams, and digitising workflows—enabling healthcare organisations to respond reactively to change, and to proactively shape strategies that anticipate future challenges, ensuring long-term sustainability and resilience.

Agile organisations, resembling living organisms, maintain a delicate balance between stability in core functions and dynamism in response mechanisms, allowing them to maintain or even improve performance under pressure (Tribe, 2017).

This study investigates how adopting an agile managerial model can enhance operational efficiency, workflow dynamics, and stakeholder satisfaction in complex healthcare settings. By examining the implementation of agile principles, the research generates valuable insights into the practical application of agility in high-stakes medical environments (Chen *et al*., 2023). The current executive structure within the Department of Paediatrics is fragmented and highly dynamic; flexibility and adaptability are essential in such a complex setting. Therefore, introducing agile principles may represent a crucial strategy for improving coordination, responsiveness, and general care delivery.

### Institute for maternal and child health–IRCCS Burlo Garofolo: the paediatric day hospital

The Paediatric Day Hospital (DH) at the Institute for Maternal and Child Health–IRCCS Burlo Garofolo in Trieste, Italy, operates within the Paediatric Department as a tertiary referral centre specialised in complex conditions, including rare diseases. It adopts a multidisciplinary, patient-centred approach by integrating diagnostics and treatments into a single-day admission model. This structure is particularly relevant for subjects outside the Friuli Venezia Giulia region, representing approximately 20% of the volume.

In 2022, the DH handled over 1,300 admissions and more than 30,000 outpatient procedures, managed by the same specialised medical and nursing team. The unit performs various diagnostic and therapeutic procedures that span multiple specialities, including endocrinology, dermatology, allergology, rheumatology, nephrology, and rare diseases. We conducted these activities within a single integrated clinical setting, where the same personnel and infrastructure manage inpatient (DH) and outpatient services. This organisational model requires careful planning and coordination, as the simultaneous delivery of services to different patient categories within a shared space necessitates flexible workflows and accurate resource allocation to maintain operational efficiency and continuity of care.

Physically, the DH is a non-surgical paediatric area comprising consulting rooms, a procedure room, and a centralised “admissions and scheduling” room. Nursing staff oversee both the intake process and the planning of future procedures.

This centralised approach optimises operational efficiency and supports care continuity by consolidating multiple assessments and interventions within a single visit.

Despite static staffing levels, the DH has consistently increased its service volumes, putting greater pressure on infrastructure and scheduling. The convergence of different service modalities within one unit presents a complex managerial setting, particularly suitable for investigating agility-related dynamics such as task coordination, workflow adaptation, and decision decentralisation in paediatric healthcare delivery.

## Materials and methods

This study adopts a case study design (Yin, 2014) to explore organisational agility within the Paediatric Day Hospital at IRCCS Burlo Garofolo. This approach is particularly suited for examining complex phenomena within their real-life context, where the boundaries between the phenomenon and its context are unclear.

A mixed-methods approach is employed to enhance the depth and validity of the findings (Creswell and Plano Clark, 2017; Johnson and Onwuegbuzie, 2004; Denzin and Lincoln, 2011). The examination integrates both qualitative and quantitative data. We gathered qualitative data through semi-structured interviews with professionals.

We collected quantitative data through two surveys: one to gather patient flow metrics, administered directly to patients, and another to assess executive agility within the Paediatric Department, based on the five characteristics of the McKinsey 5S Framework, addressed to healthcare staff.

The design of both surveys was informed by findings from the preliminary semi-structured interviews conducted with clinical staff. Thematic analysis of interview transcripts identified recurring issues related to scheduling inefficiencies, workflow bottlenecks, and communication gaps among professionals. These qualitative insights guided the development of the subsequent survey instruments.

Combining these methods allows for triangulation, providing a comprehensive understanding of organisational dynamics. This integrated approach facilitates a robust analysis of the operational structure and service delivery processes at the Paediatric Day Hospital, focussing on the 2023 patient cohort.

### Data collection

In an initial exploratory phase (August to September 2024), semi-structured interviews were conducted with healthcare staff, including seven nurses and nine doctors in the Paediatric Day Hospital. These interviews mapped existing processes and identified key organisational challenges.

We observed discrepancies between scheduled and actual patient timelines due to identified limitations in the information system. They were partly due to inconsistent data entry practices by scheduling staff, including referring physicians and nursing staff responsible for programming and managing appointment schedules, and constraints within the scheduling software. We implemented an observational survey to address this issue and capture real-time patient flow data.

### Prospective cross-sectional survey for real-time operational metrics

We conducted a cross-sectional survey between November 1 and November 30, 2024, to collect real-time patient flow, service utilisation, and operational timing data. The survey was designed to accurately measure value-added time (time directly contributing to patient care), non-value-added time (waiting or administrative delays), total cases throughput time, and identify typical patient pathways and discrepancies from scheduled times.

Insights from the semi-structured interviews allowed the research team to understand the internal organisation of the Day Hospital and map typical patient pathways. The survey was developed to capture key timing data not reliably extracted from the hospital’s information system, which presented relevant limitations and inconsistencies.

The draft version of the questionnaire was reviewed by a panel of experts – the same healthcare professionals previously interviewed – to ensure content validity and clarity of wording. Minor revisions were made following their feedback. The survey instrument was then pilot tested with a statistically significant group of patients and caregivers, who were not included in the final sample, to verify clarity and overall comprehension. Minor linguistic adjustments were made before full administration.

We administered the survey to a statistically significant sample of patients and caregivers selected from the total population of DH admissions and outpatient visits recorded in 2023. We distributed 513 questionnaires, and 443 returned, resulting in a response rate of approximately 86.4%. We calculated the sample size using power analysis, targeting a 95% confidence level with a margin of error (confidence interval) of ±5% to ensure statistical validity. We determined the sample size based on the total population of approximately 7.500 paediatric cases seen in the DH during 2023 (including admissions and outpatient services). The research board of the hospital approved the survey at the end of September 2024 [1].

The survey included the following sections:

1. Demographic Data: Collection of baseline demographic information (age, gender, and residence of patient and caregiver).
2. Visit and Service Type: Document the admission type (day hospital or outpatient), visit type (initial or follow-up), and the clinical speciality involved.
3. Timing Metrics: Detailed recording of each time point in the patient’s DH journey, from arrival at the hospital, start and end times of each consultation, periods of waiting, to final discharge and departure from the hospital. This data aimed to capture value-added versus non-value-added time, total cases throughput, typical patient pathways, and any deviations from scheduled times.
4. Patient and Caregiver Satisfaction Survey: Service quality and patient experience assessment.

### Qualitative evaluation of organisational agility

To evaluate organisational agility and workflow dynamics, we administered a questionnaire to clinical personnel at the Paediatric Day Hospital, including physicians and nursing staff. This tool assessed organisational agility within the Paediatric Department based on the five characteristics of the McKinsey 5S Framework, focussing on strategy, structure, process, people, and technology.

The draft version of this instrument was elaborated and reviewed by the authors of the study to ensure conceptual coherence and internal consistency. Construct validity was conceptually aligned with the theoretical model rather than statistically tested, as the questionnaire was explicitly designed to reflect the predefined 5S dimensions.

Consistent with the McKinsey 5S framework, we treated structure as the dimension that captures formal arrangements (e.g. hierarchy, role clarity) and the quality of inter-professional networks that enable rapid, cross-team collaboration. All questionnaire items referring to organisational networks, i.e. collaboration frequency, information flow, and interdisciplinary coordination, were loaded onto the Structure scale. The People enrolment was restricted to employee engagement, continuous learning and perceived recognition, thereby avoiding construct overlap with Structure or Processes. The questionnaire addressed role clarity, interprofessional communication, workflow flexibility, and perceived challenges in daily operations to identify key factors impacting DH services' efficiency and adaptability.

Each section of the survey consisted of items rated on a 7-point Likert scale (1 = strongly disagree, 7 = strongly agree), where higher scores indicated a greater degree of alignment with the principles of organisational agility. The maximum rating for each item reflected the full implementation of agile thinking and practices.

The instrument was pilot tested with a statistically significant group of healthcare professionals, who were not included in the main study, to confirm the clarity and interpretability of each item. Minor adjustments were made accordingly before full distribution.

### Statistical analysis

Given the ordinal nature of the data, quantitative variables were expressed as medians with interquartile ranges (IQR). We used ordinal logistic regression models to examine subgroup differences and interactions for the cross-sectional survey of day hospital patients. All *p*-values reported were two-tailed, and values < 0.05 were considered statistically significant.

Regarding the agility questionnaire, non-parametric tests assessed differences in responses across professional groups (physicians, nurses, healthcare assistants, healthcare technicians, and residents) and years of work experience at IRCCS Burlo Garofolo (<5; 5–10; 10–15; >15). When global significance was detected employing the Kruskal-Wallis test, we conducted post-hoc pairwise comparisons using Dunn's test with Bonferroni correction to adjust multiple comparisons and control for type I error.

In addition, we carried out a multivariate regression investigation to explore associations between healthcare professionals' individual and occupational characteristics and their perceptions of the agility, measured across five dimensions, basing the analysis on the total scores for each section. The multivariate model estimated the independent effect of each predictor variable (such as professional role, years of work experience, age, and gender) while adjusting for all other covariates, thereby reducing potential confounding and increasing the robustness of the results.

A significance level of *p* < 0.05 was adopted, and all estimates were reported with 95% confidence intervals.

Finally, a correlation matrix assessed the relationships among the five McKinsey 5S dimensions (strategy, structure, processes, people, technology), showing that all correlations were positive and statistically significant (0 < *r* < 1).

All statistical analyses were conducted using Stata software (version 18.5).

## Results

We reconstructed the patient journey within the Paediatric Day Hospital (DH) from interviews with hospital staff, which revealed several common challenges impacting the service’s efficiency. These aspects resulted in the survey, and we assessed operational metrics.

The nursing team responsible for scheduling used an internal digital system to consolidate diagnostic and therapeutic procedures into a single-day visit, aiming to reduce fragmentation and improve service delivery.

Despite this system, challenges emerged in aligning multiple procedures within constrained time frames. Appointment times were often set as placeholders, with the actual schedule defined during patient intake and adapted throughout the day based on physician availability and unforeseen events.

According to the patient survey, the median deviation between scheduled and actual appointments was 32 min (IQR: 9–73). Among the 1,117 procedures performed, only 9.4% occurred as scheduled, confirming the flexible and dynamic scheduling model.

Most patients arrived between 8:30 and 10:00 AM, during which the nursing staff performed primarily intake and admission activities. Although some appointments were scheduled for 8:00 AM, no procedures started until 9:00 AM.

Despite its complexity, the adaptive scheduling model proved operationally effective.

The median waiting time across all activities was 9 min (IQR: 0–25); 59% of patients waited less than 10 min, while others experienced delays exceeding two hours. The median length of stay (LOS) was 165 min (IQR: 110–238), from check-in to discharge.

Patient satisfaction was high (mean score = 4.5/5). However, greater deviation from scheduled times significantly correlated with reduced satisfaction (OR = 0.996; 95% CI: 0.993–0.999; *p* = 0.016).

In addition, information quality emerged as a key driver: more transparent communication about medical treatment increased the odds of higher satisfaction by over 15 times (OR = 15.15; 95% CI: 9.31–24.65; *p* < 0.001), while overall clarity of information was linked to an eightfold increase (OR = 7.64; 95% CI: 4.73–12.35; *p* < 0.001). Positive interactions with physicians and nurses and their courtesy and availability had a strong positive effect (OR = 12.19 and OR = 12.36, respectively; *p* < 0.001). Interestingly, total time spent in the unit did not significantly affect satisfaction levels.

### Evaluation of organisational agility

109 healthcare professionals completed the agility questionnaire, corresponding to a response rate of 75.7%. Respondents had a median age of 46 years (IQR: 35–56), and 66.1% were female. The sample included nurses (35.8%), physicians (22.0%), medical residents (18.3%), healthcare assistants (9.2%), and healthcare technicians (11.0%) Approximately 25% of respondents were employees of Burlo Hospital for over 15 years, and 17% assigned to the Paediatric Department for almost 2 decades (Table 1).

**Table 1:** Profile of healthcare personnel from the department of paediatrics

	*n* = 109
*Age, median (IQR)*	*46 (35–56)*
*Female gender, *n* (%)*	*72 (66.1%)*
*Professional qualification, n (%)*	
Nurse	39 (35.8%)
Physician	24 (22.0%)
Resident	20 (18.3%)
Healthcare assistant	10 (9.2%)
Healthcare technician staff	12 (11.0%)
Other	4 (3.7%)
*Professiona role, n (%)*	
Clinical	91 (83.5%)
Managerial	9 (8.3%)
Administrative	6 (5.5%)
Other	3 (2.7%)
*Years of work in the current department, n (%)*	
<5 years	39 (35.8%)
5–10 years	26 (23.8%)
10–15 years	28 (25.7%)
>15 years	17 (14.9%)
*Years of work at IRCCS Burlo Garofolo, n (%)*	
<5 years	33 (30.3%)
5–10 years	25 (22.9%)
10–15 years	26 (23.9%)
>15 years	25 (22.9%)
*Educational qualification, n (%)*	
Middle or high school diploma	14 (12.8%)
Bachelor's degree	19 (17.4%)
Master's degree	46 (42.2%)
Specialization/PhD	30 (27.5%)
*Years since completion of basic degree, n (%)*	
<5 years	27 (24.8%)
5–10 years	8 (7.3%)
10–15 years	20 (18.4%)
15–20 years	25 (22.9%)
>20 years	29 (26.6%)

**Note(s):** Data are presented as median (IQR) for age, and as number (percentage) for all other variables. IQR = Interquartile Range; *n*: absolute number; (%): percentage relative to the total (*n* = 109)

**Source(s):** Authors’ own work

We assessed the five McKinsey 5S dimensions using 4 to 8 items rated on a 7-point Likert scale and normalised the results to a 0–100 scale (Table 2). The resulting normalised scores were as follows: Strategy (45.8%) and Technology (40.0%) recorded the lowest levels of agility, highlighting areas where the organisation may be less responsive, integrated, or aligned with agile practices. People (72.9%) and Process (66.7%) showed intermediate levels of agility, indicating moderate support for continuous improvement, adaptability, and dynamic collaboration. Structure emerged as the highest-scoring dimension (83.3%), reflecting strong cross-functional collaboration and team integration.

**Table 2:** Median and normalised scores for each agility dimension

	Min	Max	Median	Normalised score
Strategy	8	56	30	45.8%
Structure	4	28	24	83.3%
Process	4	28	20	66.7%
People	8	56	43	72.9%
Technology	5	35	17	40.0%

**Note(s):** Min = minimum observed score; Max = maximum observed score; Median = central value; Normalised score = total score for each dimension obtained using min–max normalisation on a 0–100 scale

**Source(s):** Authors’ own work

#### Strategy

Staff perceptions of strategic alignment were relatively low. The median score for clarity of mission and vision was 2 (IQR: 2–4), and strategic objectives were also poorly communicated (median = 3; IQR: 2–4). Staff involvement in goal setting was limited (median = 3; IQR: 3–5), though professionals acknowledged their contribution to overall outcomes (median = 5; IQR: 3–6). Medical doctors reported higher perceived contribution to strategic objectives (median = 5; IQR: 3–6), while medical residents reported significantly lower engagement (median = 3; IQR: 2–5; *p* = 0.004; Table 3).

**Table 3:** Median (IQR) agility scores by professional qualification and years of work

	Strategy	Structure	Process	People	Technology
*Total score, median (IQR)*	*30 (19–36)*	*24 (20–24)*	*20 (17–24)*	*43 (37–48)*	*17 (11–20)*
*Professional qualification, median (IQR)*					
Nurse	31 (16–37)	23 (20–34)	20 (17–22)	40 (38–47)	15 (10–19)
Physician	34 (23–37)	24 (21–26)	22 (18–34)	48 (35–48)	18 (13–21)
Medical resident	22 (14–33)	24 (21–24)	20 (15–24)	44 (30–47)	16 (11–21)
Healthcare assistant	23 (18–26)	20 (20–24)	19 (16–20)	38 (34–40)	16 (10–19)
Healthcare technician staff	32 (26–39)	24 (21–24)	22 (20–24)	47 (42–48)	21 (20–24)
*Years of work at Burlo, median (IQR)*					
<5 years	30 (16–35)	24 (21–24)	20 (16–24)	46 (38–48)	17 (11–21)
5–10 years	30 (24–37)	24 (20–24)	21 (19–24)	48 (39–48)	19 (18–23)
10–15 years	24 (16–34)	24 (21–24)	20 (17–24)	42 (37–48)	15 (12–17)
>15 years	31 (16–41)	22 (20–24)	20 (14–22)	40 (31–45)	11 (7–19)

**Note(s):** Scores for each agility dimension are reported by professional qualification and by years of work at IRCCS Burlo Garofolo. Data are presented as median (IQR); IQR = Interquartile Range

**Source(s):** Authors’ own work

#### Structure

Collaboration across disciplines was strong. Professionals frequently worked together to manage clinical issues (median = 6; IQR: 5–6), with physicians reporting the highest levels of team integration (median = 6; IQR: 6–7) and healthcare assistants scoring lower (median = 5; IQR: 5–6; *p* = 0.017). Despite interdisciplinary engagement, internal communication within the same professional category was less robust (median = 4; IQR: 3–5).

#### Process

Participants indicated having sufficient autonomy to make independent clinical and managerial decisions, when necessary, with a median score of 6 (IQR: 5–6). We regularly reviewed the care processes based on patient feedback and adjusted to incorporate evidence-based best practices, receiving a median score of 5 (IQR = 4–6). However, significant differences (*p* = 0.020) emerged across professional roles (Table 3).

#### People

Staff members declared high levels of motivation and adaptability. Most expressed pride in working at the hospital (median = 6; IQR: 5–6) and commitment to updating clinical knowledge (median = 6; IQR: 5–6). Staff with fewer than 5 years at the hospital reported higher engagement in professional development (median = 6; IQR: 6–7) than those with 11–15 (median = 6; IQR: 5–6) or over 15 years (median = 5; IQR: 5–6; *p* = 0.003). Doctors were more inclined to propose innovative solutions (median = 6; IQR: 5–6), while medical residents and healthcare assistants reported significantly lower scores (median = 4; IQR: 4–5; *p* = 0.001). Perceptions of institutional recognition were slightly less favourable (median = 5; IQR: 4–6), with notable variation across roles (*p* = 0.012).

#### Technology

Technology emerged as the least favourable dimension of agility. While the use of new technologies to enhance the patient care pathway was rated moderately (median = 4; IQR = 2–5), perceptions varied significantly according to staff tenure (*p* < 0.001). Professionals with 5–10 years of experience at Burlo expressed greater agreement that the hospital quickly adopts technologies with proven patient benefits (median = 5; IQR = 4–5) compared to colleagues with 11–15 years (median = 3; IQR = 2–5) and over 15 years of service (median = 3; IQR = 1–4).

The hospital’s digital infrastructure was generally perceived as non-intuitive and difficult to use (median = 3; IQR = 2–4). Similarly, we described information systems and digital tools as only marginally helpful for daily workflows (median = 3; IQR = 2–4).

Technological readiness also varied by professional group. Healthcare technicians felt better trained to use digital tools (median = 5; IQR = 5–6) compared to nurses (median = 3; IQR = 2–5), with a statistically significant difference (Table 3; *p* = 0.012).

Staff tenure influenced perceived digital competence (*p* = 0.005). Professionals with more than 15 years of service reported greater difficulty using available technologies (median = 3; IQR = 1–4), while those with 5–10 years of experience felt more confident and prepared (median = 5; IQR = 4–5).

### Correlation matrix between the five dimensions of agility

**Figure 1:**
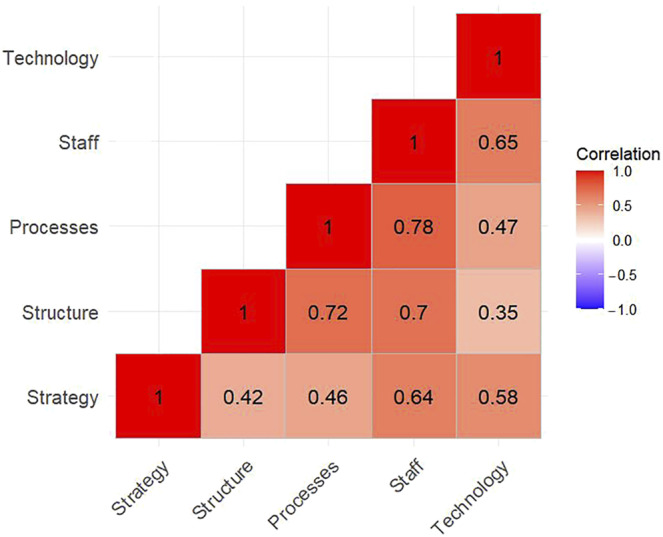
Correlation matrix among the five dimensions; all correlations are positive and statistically significant (0 < *r* < 1)

We examined the relationships among the five McKinsey 5S dimensions (Figure 1) with a correlation matrix. All correlations were positive and statistically significant (0 < *r* < 1), confirming consistent interdependencies across the framework. Strategy is strongly associated with People (*r* = 0.64) and has a moderate link with Structure (*r* = 0.42), suggesting that strategic vision is connected to staff engagement and organisational architecture. Structure was strongly correlated with Process (*r* = 0.72) and People (*r* = 0.70), reflecting the close interplay between teamwork, operational workflows, and organisational design. Process also demonstrated a high interaction with People (*r* = 0.78). At the same time, moderate connections emerged between Process and Technology (*r* = 0.47) and Technology and People (*r* = 0.65), indicating that digital enablers align most closely with staff dynamics and workflow responsiveness.

### Multivariate model

We performed a multivariate regression analysis across the five dimensions to evaluate how demographic and professional variables impact perceived agility (Table 4). We evaluated the effect of an independent variable holding all other variables constant. Gender did not significantly affect any of the assessed dimensions *We adopted a* significance level of *p* < 0.05 and reported all estimates with 95% confidence intervals.

**Table 4:** The multivariate model

	Strategy		Structure		Process		People		Technology	
	Coeff	*p*-value	Coeff	*p*-value	Coeff	*p*-value	Coeff	*p*-value	Coeff	*p*-value
*Professional qualification (ref. Nurse)*										
Physician	3.38	0.292	2.79	*0.020*	4.86	*0.001*	3.70	0.140	2.44	0.159
Medical resident	−10.37	0.070	3.77	0.075	4.06	0.109	−2.10	0.636	2.37	0.439
Healthcare assistant	−7.82	*0.041*	−1.81	0.197	−2.01	0.231	−6.23	*0.037*	−0.50	0.804
Healthcare technician staff	−0.04	0.992	2.43	0.120	3.88	*0.040*	1.87	0.568	6.11	*0.008*
*Years of work at Burlo (ref. <5* *years)*										
5–10 years	−9.23	*0.034*	−2.36	0.139	−4.49	*0.020*	−6.58	0.052	−0.98	0.672
11–15 years	−16.02	*0.001*	−1.41	0.429	−5.14	*0.017*	−9.09	*0.017*	−5.22	*0.046*
>15 years	−15.49	*0.004*	−4.50	*0.023*	−9.15	*<0.001*	−16.71	*<0.001*	−8.89	*0.002*
Age	0.38	0.080	0.22	*0.005*	0.40	*<0.001*	0.39	*0.020*	0.27	*0.021*
Gender	2.85	0.237	−0.86	0.334	−0.14	0.894	0.99	0.596	0.82	0.527

**Note(s):** The multivariate model evaluates the effects of professional qualification (reference category: nurse), years of work at Burlo Garofolo Hospital (reference category: <5 years), age, and gender on the five dimensions of agility. A significance level of *p* < 0.05 was adopted, and all estimates are reported with 95% confidence intervals

**Source(s):** Authors’ own work

#### Strategy

Compared to nurses (reference group), healthcare assistants registered significantly lower strategic scores (Coeff. = −7.82, *p* = 0.041). Professionals with longer work experience at the IRCCS Burlo Garofolo exhibited significantly lower perceptions of alignment.

#### Structure

Physicians perceived higher levels of interdisciplinary collaboration than nurses (Coeff. = 2.79, *p* = 0.020). Staff with >15 years at the hospital indicated lower structural agility (Coeff. = −4.50, *p* = 0.023). Interestingly, age was positively associated with this dimension (Coeff. = 0.22, *p* = 0.005).

#### Process

Significantly higher process scores were observed among physicians (*Coeff.* = 4.86; *p* = 0.001) and technicians (*Coeff.* = 3.88; *p* = 0.040), revealing greater agreement with the notion that processes are rapid, iterative, and regularly reviewed. Conversely, longer work experience was negatively associated with this dimension, with significantly lower scores observed across all experience categories beyond five years, particularly among those with more than 15 years of service (Coeff. = −9.15, *p* < 0.001). Notably, however, the score increased by 0.40 points for each additional year of age (Coeff. = 0.40, *p* < 0.001).

#### People

Lower scores were documented by healthcare assistants (Coeff. = −6.23, *p* = 0.037) and staff with 11–15 years (Coeff. = −9.09, *p* = 0.017) or more than 15 years of work experience (Coeff. = −16.71, *p* < 0.001). In contrast, age was positively associated with this dimension (Coeff. = 0.39, *p* = 0.020), reinforcing a recurring trend across several dimensions: older staff tended to display a more dynamic and collaborative attitude within their teams, regardless of their professional role, length of service, gender, or job classification.

#### Technology

A significantly higher perception of the accessibility and functionality of available technologies within the unit was among healthcare technical staff (Coeff. = 6.11, *p* = 0.008). In contrast, longer work experience at the Burlo Paediatric Day Hospital was negatively associated with this dimension: the 11–15-year group (Coeff. = −5.22, *p* = 0.046) and the >15-year group (Coeff. = −8.89, *p* = 0.002) noted significantly lower scores. Age was positively associated with perceptions of technology (Coeff. = 0.27, *p* = 0.021), further underlining a consistent pattern across the data.

## Discussion

This study explored organisational agility within the Paediatric Day Hospital (DH) of IRCCS Burlo Garofolo, applying the McKinsey 5S Framework to evaluate five key dimensions: strategy, structure, process, people, and technology. Findings highlight strengths and areas for improvement in operational agility, reflecting broader challenges and opportunities within public-sector paediatric healthcare.

The results reinforce what existing literature has suggested but not thoroughly examined: agility in healthcare can enhance responsiveness, collaboration, and satisfaction, but its implementation is uneven and context-dependent (Nanji *et al*., 2018; Ribeiro and Domingues, 2018). The Paediatric Day Hospital represents a highly complex environment that deals with rare and multi-speciality paediatric conditions. Flexible scheduling, interdisciplinary teams, and patient-centred coordination are essential in this setting.

Agility scores varied across dimensions. Structure emerged as the most agile domain, reflecting the collaborative and team-based nature of the DH’s operations. This outcome aligns with the unit’s intentionally non-hierarchical and loosely structured organisation, which requires continuous professional coordination. High scores in interdisciplinary collaboration confirm that flexible arrangements support shared decision-making and adaptive care.

However, internal communication within professional groups was less robust. Physicians reported stronger perceptions of collaboration than nurses, while staff with more than 15 years of service expressed lower structural agility, possibly reflecting fatigue or scepticism toward past organisational changes.

The Process dimension showed moderate agility. Staff reported autonomy in making clinical and managerial decisions, and care pathways were regularly reassessed to meet evolving patient needs. Notably, the strong correlation between processes and people emphasises the vital role of engaged, empathetic staff in driving fast, iterative workflows that adapt to complexity. As noted in the literature (Radnor and Osborne, 2013), response variations suggest that professional background influences process agility. Physicians and technicians scored higher on this dimension, whereas longer-tenured professionals tended to perceive processes as more rigid and less adaptable over time.

People were the second-highest rated dimension, with most professionals expressing pride, adaptability, and a commitment to ongoing learning. Nevertheless, lower scores were reported by healthcare assistants and staff with over 15 years of service, possibly pointing to reduced motivation or recognition. These findings may imply that, over time, healthcare professionals working in the unit may experience a decline in engagement, which could limit their participation in improvement efforts. This trend highlights the need for inclusive strategies that value institutional memory and integrate experienced staff into agile transformation efforts.

Strategy emerged as a weaker dimension. Professionals perceived limited clarity in the hospital’s mission and strategic goals, with minimal involvement in goal-setting activities. Healthcare assistants reported significantly lower strategic alignment than nurses, and scores consistently dropped with increasing years of service. These patterns suggest disconnecting from the organisational vision among more experienced staff members. According to Denning (2018), agile organisations require a clearly articulated “North Star” to guide decisions and align teams, which deserves focused managerial attention.

It echoes what scholars have highlighted: strategic ambiguity and limited stakeholder engagement can weaken alignment and compromise performance (Goldman *et al*., 1995; Ribeiro and Domingues, 2018).

Technology was the least favourable domain, highlighting persistent issues in usability and integration. While the DH setting demands flexible scheduling and rapid coordination, many respondents cited a lack of training and inflexible and unintuitive digital systems as barriers. These findings echo prior studies (Unterhofer *et al*., 2021), highlighting gaps like low data interoperability and uneven adoption of new tools. The fragmented and unstructured nature of scheduling workflows stems partly from these limitations, suggesting a misalignment between technological investments and frontline operational needs. Healthcare technicians felt more confident using digital tools, while nurses and long-tenured staff reported greater difficulties, reinforcing concerns about unequal support during digital transitions.

From a dynamic capabilities perspective, agility depends on an organization’s ability to sense, adapt, and reconfigure (Teece, 2014). The correlation analysis revealed strong interdependencies among people, structure, and process, indicating that staff engagement and coordination drive the DH’s adaptive potential. In particular, the substantial interconnection between *processes* and *people* underscores the critical role of passionate and responsive staff in enabling fast, iterative workflows that can flexibly adapt to patients' needs.

Structure also played a central role in agility dynamics, with high correlations found between structure and process and structure and people, emphasising how collaborative networks and effective teamwork support workflow adaptation and staff motivation. A firm correlation between strategy and people further emphasises the importance of an articulated strategic vision: when employees understand and trust the organisational direction, they are more likely to feel empowered, take initiative, and align their efforts with shared goals. Similarly, the moderate association between strategy and structure suggests that strategic clarity facilitates interprofessional coordination across formal boundaries.

Technology showed moderate correlations with process and people, suggesting that when properly implemented, digital tools can support workflow evolution and empower teams.

The multivariate model adds nuance to this picture, demonstrating that perceptions of agility vary by role, tenure, and age. Physicians and technicians consistently rated agility higher, particularly in process and technology domains.

Observing that agility scores tend to decrease with increasing years of experience suggests an important reflection. It is plausible that long-tenured professionals, having greater exposure to organisational dynamics and past reforms, develop a more critical view of organisational inefficiencies. This phenomenon might also reflect fatigue or disengagement stemming from repeated changes perceived as top-down or insufficiently inclusive. Organisational changes often face resistance from experienced staff who have witnessed multiple reform cycles with limited success. Rather than interpreting this as mere resistance to change, it may represent valuable institutional memory and critical awareness of implementation challenges that we should leverage in the change process (Fatima and Fatima, 2022).

Taken together, these findings confirm that agility in healthcare is multifaceted. While flexible workflows and strong interpersonal collaboration are clear strengths, strategic clarity, digital usability, and inclusive change processes remain areas of development. The DH model—centred on minimising patient burden by concentrating care into a single day—requires constant adaptation, reinforcing the importance of structural and human enablers to sustain agile operations.

### Theoretical contributions

This study expands the literature on organisational agility by empirically examining its application within a public-sector paediatric healthcare context, an area that remains relatively underexplored (Tolf *et al*., 2015; Ahmad and Wasim, 2023; Jaafar *et al*., 2025). While prior research has primarily focused on agility in IT or emergency-response scenarios (Alotaibi and Almudhi, 2023; Nanji *et al*., 2018), this study broadens the analytical scope by evaluating agile behaviours in routine care delivery, particularly in settings marked by complex diagnostic and therapeutic coordination.

A central theoretical advancement lies in extending the McKinsey 5S Framework beyond traditional corporate environments. By applying it to specialised clinical organizations, such as a paediatric Day Hospital, the framework offers a multidimensional lens through which we can assess agility across strategy, structure, process, people, and technology. It addresses the literature for more robust and context-sensitive frameworks to evaluate agile performance in healthcare teams (Alotaibi and Almudhi, 2023; Ribeiro and Domingues, 2018). It also reveals how agility dimensions manifest differently in clinical versus conventional business settings.

Furthermore, the Day Hospital case illustrates the emergence of agile practices from below, guided not by formal strategies but by the ability to respond on the front lines to changing clinical needs. This phenomenon challenges dominant top-down models of agility implementation and suggests that organic, context-sensitive adaptation may be a more fitting paradigm in highly specialised healthcare environments.

Finally, the findings reinforce theoretical claims about the compatibility between Lean and Agile paradigms (Tolf *et al*., 2015). The coexistence of Lean-based efficiency, achieved through integrated scheduling and coordinated processes and Agile-enabled responsiveness, visible in dynamic decision-making and interprofessional collaboration, demonstrates how hybrid models can support adaptability in unpredictable clinical conditions.

### Managerial and social implications

This study findings offer valuable guidance for healthcare managers working in complex and resource-limited environments. One of the fundamental challenges identified was the low level of strategic alignment, with staff often feeling disconnected from the organisation's broader goals. This condition implies the need for managers to actively involve frontline professionals in developing strategic priorities, ensuring that these are communicated and reflect everyday clinical realities. When staff understand and contribute to the hospital’s direction, they are more likely to be engaged and responsive.

Technology also emerged as a significant obstacle to agility. Many professionals reported difficulties using digital systems due to poor usability, limited training, and a lack of integration with their workflows. Managers should prioritise improving digital infrastructure while adopting co-design approaches that involve clinical staff directly in choosing and adapting digital systems. This approach ensures practical relevance and builds user ownership and confidence.

While interdisciplinary collaboration was intense, communication within professional groups was less consistent. Creating spaces and routines for internal dialogue—such as peer briefings or shared decision platforms—could strengthen coordination and reduce bottlenecks within roles.

At an organisational level, managers face the challenge of balancing formal hierarchies—needed to uphold clinical standards—with room for autonomy so that multidisciplinary teams can respond quickly to patient needs. The Day Hospital model reveals that flexible structures can coexist with rigorous protocols, provided that decision-making mechanisms are in place to support both responsiveness and accountability.

Finally, we should not consider experienced staff as resistant to change but as valuable assets Their deep understanding of institutional dynamics can inform more effective reform strategies. Managers could involve them as internal consultants or change agents, leveraging their insights to design and implement practices grounded in operational reality.

Beyond managerial strategies, these findings also carry important social implications for paediatric healthcare organisations. In settings such as DH, where clinical activities follow tightly scheduled workflows, structural inefficiencies and fragmented communication may hinder the care experience. Adopting agile organisational models can help overcome these constraints by fostering greater adaptability, decentralised decision-making, and responsiveness to emergent patient needs. However, as this study was conducted within a semi-autonomous ambulatory care unit, the implications mainly concern comparable outpatient environments rather than acute inpatient settings, where systemic interdependencies and emergency pressures could require different agility configurations.

When embedded effectively, agility becomes a tool not only for operational refinement but for improving the lived experience of care.

Patient satisfaction, particularly in paediatric contexts, hinges on family perceptions, emotional engagement, and the quality of communication with clinical staff. An agile environment, capable of balancing standardised processes with customisable interventions, can enable healthcare providers to better accommodate individual preferences, leading to improved adherence to treatment plans and enhanced therapeutic relationships (Manary *et al*., 2013).

Organisational agility thus emerges as a strategic lever through which healthcare institutions may translate managerial adjustments into tangible improvements in patient-centred outcomes. Future research could build on these insights by incorporating outcome indicators, such as adherence metrics and recovery trajectories, to empirically validate the long-term impact of agility on care effectiveness and system-level performance.

## Conclusions and limitations

This study demonstrates that organisational agility can emerge organically in specialised healthcare settings, even without explicit agile strategies. Despite this encouraging evidence, we should interpret these findings as context-bound rather than universally transferable. The analysis reflects a single tertiary Italian paediatric referral centre, observed over one month and specialising in rare disease pathways. Organisational culture, regulatory frameworks, and case mix can differ in community hospitals, adult services or private-sector providers. Moreover, as the investigated DH operates as a relatively closed ambulatory care system, its workflows are only marginally affected by external contingencies such as emergency admissions or acute deteriorations occurring in other hospital units.

Accordingly, our results constitute testable propositions that require replication through multi-site and longitudinal designs before making broader claims about “organic” agility in health care.

Applying the McKinsey 5S Framework, the research highlights that agility is not a single concept but a configuration of interdependent elements, each influenced by professional roles, staff tenure, infrastructure, and institutional culture.

Notable strengths emerged in team collaboration, autonomy in decision-making, and staff motivation. However, strategic alignment and technological readiness remain areas of concern, particularly among long-serving personnel not yet fully integrated into digital transformation efforts.

Addressing these gaps while delivering high-quality, patient-centred care in increasingly complex and dynamic environments can strengthen healthcare organisations' adaptive capacity.

Despite the comprehensive mixed-methods approach, this study acknowledges several methodological limitations. First, self-reported data from surveys and interviews may introduce response bias, as participants might provide socially desirable answers influenced by organisational power dynamics (Creswell and Plano Clark, 2017). Second, the one-month observational period may not capture seasonal variations in patient flow and staff workload that could affect perceptions of organisational agility. Third, while statistically significant, the sample represents only a subset of the patient and staff population, potentially limiting generalisability (Yin, 2014). Fourth, as a single-institution study conducted in a specialised paediatric referral centre operating as a semi-autonomous ambulatory care unit, the findings may have limited applicability to healthcare settings with different organisational structures, clinical missions, or systemic interdependencies typical of broader hospital networks. (Radnor *et al*., 2012). Finally, the cross-sectional design prevents evaluating how agile capabilities develop over time or their long-term effects on organisational performance.

## Ethical statement

The present study was conducted following the principles of the Declaration of Helsinki and was approved by the Institutional Review Board of the Institute for Maternal and Child Health – IRCCS “Burlo Garofolo” (Protocol No. GEN/INT 0000615–11/03/2025). We obtained written informed consent from all study participants.

## Note

1. All procedures adhered to the Declaration of Helsinki and EU GDPR 2016/679 requirements. Data were pseudonymised at source, stored on encrypted drives accessible only to the research team, and reported in aggregate to preclude re-identification.
